# The relationship between the ratio of the supraspinatus muscle thickness measured by ultrasound imaging and glenohumeral subluxation in stroke patients: a cross-sectional study

**DOI:** 10.3389/fneur.2024.1407638

**Published:** 2024-08-23

**Authors:** Hualong Xie, Qing Zhang, Jiawen Zhan, Jige Dong, Jing Chen, Guoxin Kang, Huilin Liu, Qiuchen Huang, Liguo Zhu, Ko Onoda, Hitoshi Maruyama, Shan Liu, Ming Huo

**Affiliations:** ^1^Wangjing Hospital of China Academy of Chinese Medical Sciences, Beijing, China; ^2^The Affiliated Hospital of Inner Mongolia Medical University, Hohhot, China; ^3^Beijing Chaoyang Integrative Medicine Emergency Medical Center, Beijing, China; ^4^China Rehabilitation Research Center, Beijing, China; ^5^Department of Physical Therapy, International University of Health and Welfare, Otawara, Japan; ^6^School of Rehabilitation Sciences and Engineering, University of Health and Rehabilitation Sciences, Qingdao, China; ^7^Jilin Province Power Hospital, Changchun, China; ^8^Shaanxi Provincial Rehabilitation Hospital, Xi’an, China

**Keywords:** glenohumeral subluxation, ultrasound imaging, supraspinatus muscles thickness, stroke, hemiplegia

## Abstract

**Introduction:**

Glenohumeral subluxation (GHS) is a common complication in stroke patients with hemiplegia, occurring in approximately 17–81% of cases. This study aims to evaluate the relationship between shoulder muscle thickness and the degree of subluxation using ultrasound imaging.

**Methods:**

A cross-sectional study of 61 stroke patients with hemiplegia was conducted, measuring supraspinatus muscle thickness, deltoid muscle thickness, and acromion-greater tuberosity (AGT). Logistic regression and ROC analyses were used. ROC curves, calibration plots, and decision curves were drawn on the training and validation sets.

**Results:**

According to logistic regression analysis, the ratio of supraspinatus muscle thickness was statistically significant (OR: 0.80; 95% CI: 0.70–0.92; *p* < 0.01), and it was an independent factor for evaluating the presence or absence of GHS. An AUC of 0.906 (95% CI, 0.802–1.000) was found in the training set; meanwhile, the AUC in the validation set was 0.857 (95% CI, 0.669–1.000), indicating good performance. According to the training set ROC curve, the most effective statistical threshold was 93%, with a sensitivity of 84% and a specificity of 96%.

**Discussion:**

The ratio of supraspinatus muscle thickness is a valuable criterion for evaluating GHS risk, supporting targeted rehabilitation interventions.

## 1 Introduction

Stroke is a major health problem worldwide. Since 2015, stroke, as a chronic non-communicable disease, has become the leading cause of death and disability in China, posing a serious threat to citizens’ health (1–3). The onset of a stroke causes paralysis in half of the body, somatosensory disorder and muscle weakness are associated with decreased balance and walking ability, as well as increased movement restriction in daily life (4–6). Glenohumeral subluxation (GHS) is a relatively common complication in patients with hemiplegia, occurring in approximately 17–81% of patients with cerebrovascular disorder hemiplegia (with 73% of these cases occurring in the acute stage) (7–9). Such differences in prevalence are mainly due to differences in measurement methods and time of onset (10–12). At a 10-month follow-up, 67% of patients diagnosed with cerebrovascular disorder hemiplegia demonstrated limited functional recovery—notably, this limited recovery may be due to GHS (13). GHS is defined as partial or incomplete dislocation due to changes in the joint’s mechanical integrity (14). GHS not only causes shoulder pain, seriously affects upper limb function, and limits activities of daily living, but also negatively impacts follow-up rehabilitation (15, 16). Therefore, early treatment of subluxation is essential to prevent further deterioration into a state that is difficult to correct (17).

Shoulder stability is dependent on the muscle and ligament structure, as opposed to skeletal morphology. Reduced muscle strength around the shoulder joint can cause sub-dislocation of the shoulder (18, 19). The supraspinatus and posterior deltoid muscles are the most important muscles for maintaining shoulder stability (20, 21). The supraspinatus is associated with shoulder impingement, periarthritis shoulder, shoulder dysfunction, and hemiplegia (22, 23). Previous studies have stated that palpation methods used to evaluate subluxation of the shoulder joint are less sensitive when detecting early signs of GHS and mild subluxation associated with severe paralysis (24). Although roentgenographic evaluation is objective, reliable, and effective (25, 26), it is generally not used, and its clinical usefulness is questioned because of cost, time, and risk of radiation exposure. Thus, it is not recommended for the clinical evaluation of GHS (27).

In recent years, ultrasound imaging has been used to evaluate the degree of subluxation of the shoulder joint. By measuring the acromion-greater tuberosity (AGT) distance, the presence of subluxation in hemiplegic patients can be determined. It has been reported that dislocation can be evaluated, suggesting that subluxation occurs when the difference between the affected and unaffected sides is 0.2 cm or wider (12). Measurement of the AGT distance using ultrasound imaging has been found to have adequate intra- and inter-rater reliability. It has also been shown to be very reliable compared with experienced and beginner evaluators (28, 29). Previous studies have also reported that in patients with hemiplegic cerebrovascular injury, supraspinatus muscle thickness, and deltoid muscle thickness measurements are highly reliable at each angle of abduction of the shoulder joint (30, 31). In a previous study, one examiner measured the supraspinatus muscle thickness of 20 healthy subjects twice, while another examiner performed the same procedure a few days later. The measurement was found to be reliable for each examiner’s measurements, as well as between examiners (32). Thus, the intra- and inter-rater reliability of supraspinatus thickness measurement is considered to be high. The supraspinatus muscle plays a crucial role in maintaining shoulder joint stability and function. Evaluating the structure and function of the supraspinatus muscle can clarify the risk factors for shoulder movement abnormalities and subluxation, providing accurate guidance for clinical interventions. Notably, ultrasounds can be used to effectively evaluate the supraspinatus muscle in the assessment of shoulder subluxation (18). Based on the above, we consider that there is a correlation between the degree of shoulder subluxation and the thickness ratio of the supraspinatus muscle and use ultrasound to explore this connection.

The aim of this study is to use ultrasound imaging to elucidate the relationship between the muscles around the shoulder joint and the degree of subluxation. Determine the association the degree of subluxation and the ratio of supraspinatus muscle thickness shall be determined, and the validity of the evaluation of the degree of subluxation shall be examined.

## 2 Materials and methods

### 2.1 Participants and method

This cross-sectional study was conducted in a hospital department of physical therapy between March and November 2020. Participants included 61 stroke patients with hemiplegia. The inclusion criteria were as follows: First occurrence of stroke with hemiplegia; patients with onset time within 6 months; age greater than 18 years old; being able to maintain a sitting position alone. The exclusion criteria were as follows: unstable vital signs; dementia and mental illness; brain stem lesion; a disease of both sides lesion; serious respiratory system disease, cardiovascular disease, and other severe comorbidities; shoulder pathology or recent surgery to the neck, arm, or shoulder; and those unable to volunteer for any reason. The participants’ AGT of both affected and unaffected sides was measured. If the AGT of the unaffected side minus the AGT of the affected side was 0.2 cm or wider, they were considered to have GHS (12). In accordance with the Declaration of the World Medical Association, the study design was approved by the committee on research ethics at the institution where the research was conducted. Informed consent was obtained from all patients in the study and/or their legal guardians.

The measurements were supraspinatus muscle thickness, deltoid muscle thickness, and AGT by ultrasound imaging. Bilateral supraspinatus muscle thickness and AGT were measured twice in all patients, and the average value was used as the target value.

The ratio of supraspinatus muscle thickness was calculated as follows: Supraspinatus muscle thickness ratio (%) = Supraspinatus muscle thickness on the affected side/supraspinatus muscle thickness on the unaffected side × 100%.

The ratio of deltoid muscle thickness was calculated as follows: Deltoid muscle thickness ratio (%) = Deltoid muscle thickness on the affected side/deltoid muscle thickness on the unaffected side × 100%.

The parameters were measured using an ultrasound diagnostic system (Sonosite180 plus, United States) in combination with a 7.5 MHz linear transducer. All measurements were performed by the same physical therapist.

### 2.2 Measurement site and method

During the measurement of the parameters, each patient was placed in a standard position: shoulder in neutral rotation, elbow at 90° of flexion, and forearm in pronation. The forearms were rested on a pillow (placed on the patient’s lap), but the elbow remained unsupported to ensure that the shoulder girdle was not elevated. The researcher placed the affected arm in this standardized position and ensured that the position was maintained during the period of measurement. When measuring the supraspinatus muscle, the researcher measured the corresponding limb from the patient’s back. The probe was placed on the measurement site, the center point of the scapular spine on the measurement side with respect to the supraspinatus muscle. The probe was placed perpendicular to the long axis of the supraspinatus muscle. We then identified the cross-section of the supraspinatus, froze the image, and measured the distance of the supraspinatus thickness. To measure deltoid muscle thickness, the probe was placed perpendicular to the longitudinal axis of the humerus at the midpoint of the joint between the lateral acromion edge and deltoid tuberosity. Next, we identified the cross-section of the deltoid muscle, froze the image, and measured the distance of the deltoid muscle thickness (31).

**Figure 1:**
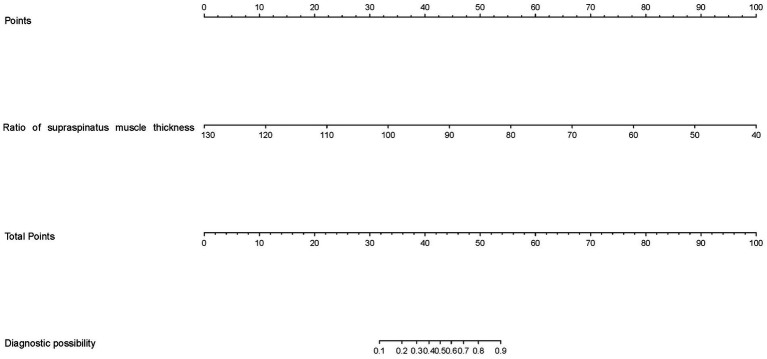
The constructed nomogram for predicting GHS.

The AGT was measured between the lateral edge of the acromial process and the upper edge of the greater tubercle of the humerus. With the arm, elbow, and shoulder in the standardized position, the ultrasonic probe was placed on the lateral edge of the acromion along the longitudinal axis/longitudinal axis of the humerus to scan the shoulder joint. When the lateral edge of the acromion and the upper edge of the greater tubercle appeared on the screen at the same time, the images were frozen, and the AGT was measured (Figure 1).

### 2.3 Statistical analysis

Measurement data were subjected to normal distribution tests. When the data conformed to a normal distribution, they were described using the mean ± standard deviation. Paired and independent sample t-tests were used to evaluate differences. When the data did not conform to a normal distribution, Mann–Whitney U tests and Wilcoxon signed-rank tests were used to evaluate differences. Count data were described using percentages, and chi-square tests were used. Univariate and multivariate logistic regression analyses were used to analyze factors related to the presence or absence of GHS. The applicability of logistic regression analysis was determined using the Hosmer-Lemeshow test. ROC curves, calibration plots, and decision curves were drawn on the training and validation sets to analyze and evaluate their discriminatory ability, predictive accuracy, and clinical utility. Statistical analysis was performed using SPSS version 23 and R software version 4.1.3. Significance was set at *p* < 0.05.

## 3 Results

The participant consisted of 61 patients with cerebrovascular accident hemiplegia (right-side hemiplegia: 29 patients, left-side hemiplegia: 32 patients; Brunnstrom stage I: 18 patients, stage II: 10 patients, stage III: nine patients, stage IV: 10 patients, stage V: 11 patients, and stage VI: three patients). Of the 61 participants, 35 patients had GHS (57%). The participants’ AGT of both the affected side and unaffected side were measured; if the AGT of the unaffected side minus the AGT of the affected side was 0.2 cm or more, they were considered to have subluxation (12). The participants’ characteristics are described in detail in Table 1.

**Table 1:** Participants’ characteristics.

	All participants (*n* = 61)	Group with GHS (*n* = 35)	Group without GHS (*n* = 26)	*p* values
Age (year), Mean ± SD	56.4 ± 13.2	59.6 ± 13.8	53.9 ± 12.3	*p* > 0.05
Height (cm), Mean ± SD	170.6 ± 7.0	170.4 ± 5.8	170.8 ± 7.9	*p* > 0.05
Weight (kg), Mean ± SD	75.5 ± 14.5	76.2 ± 15.9	74.9 ± 13.5	*p* > 0.05
Gender, *n*	Male: 50			
	Female: 11			
Affected side, *n*	Right: 29			
	Left: 32			
Time of onset, days	96			

Data are reported as the mean ± standard deviation unless otherwise indicated.

The results of the paired samples *t*-test showed that in the group with GHS, the supraspinatus muscle thickness on the affected side was significantly smaller than that on the unaffected side. The comparison between the thickness of the deltoid muscle on the affected and the unaffected sides showed statistical significance in both the group with GHS and the group without GHS. In both groups, the thickness of the deltoid muscle on the affected side was significantly reduced compared to the thickness of the supraspinatus muscle on the unaffected side. Furthermore, the independent sample *t*-test showed that the thickness of the affected side supraspinatus muscle was statistically significant between the group with GHS and the group without GHS, with a significant decrease in thickness in the group with GHS. The ratio of supraspinatus muscle thickness was statistically significant between the groups with and without GHS, with a significant decrease in the latter (Table 2). The Pearson correlation coefficient between the ratio of supraspinatus muscle thickness and the ratio of deltoid muscle thickness was also analyzed; the correlation coefficient was *r* = 0.269 (*p* < 0.05), indicating a correlation.

**Table 2:** Measurement results with and without subluxation.

		Group with GHS (*n* = 35)	Group without GHS (*n* = 26)	*p* values for comparison between groups
Supraspinatus muscle thickness (cm)	Unaffected sides	1.9 ± 0.3	1.9 ± 0.3	*p* > 0.05
	Affected sides	1.6 ± 0.3	1.8 ± 0.2	*p* < 0.01
	*p* values	*p* < 0.01	*p* > 0.05	
Deltoid muscle thickness (cm)	Unaffected sides	1.9 ± 0.4	2.0 ± 0.4	*p* > 0.05
	Affected sides	1.7 ± 0.4	1.8 ± 0.4	*p* > 0.05
	*p* values	*p* < 0.01	*p* < 0.01	
Ratio of deltoid muscle thickness (%)		86.5 ± 15.8	91.4 ± 16.1	*p* > 0.05
Ratio of supraspinatus muscle thickness (%)		81.6 ± 15.7	97.5 ± 9.9	*p* < 0.01

Data reported as the mean ± standard deviation unless otherwise indicated.

We randomly divided 61 samples into a training set (*n* = 43) and a verification set (*n* = 18) at a ratio of 70–30%. All data conformed to a normal distribution. There were no significant differences in age, height, weight, supraspinatus muscle thickness ratio, and deltoid muscle thickness ratio between the training set and the validation set (*p* > 0.05) (Table 3). In the training set, there were no significant differences in height, age, weight, or deltoid muscle thickness ratio (*p* > 0.05); however, a statistically significant difference was observed for the supraspinatus muscle thickness ratio (*p* < 0.01) (Table 4).

**Table 3:** Balance test between training set and validation set.

Variables	Total (*n* = 61)	Training sets (*n* = 43)	Validation sets (*n* = 18)	Statistic	*p*
Age (year), Mean ± SD	56.36 ± 13.19	55.51 ± 13.24	58.39 ± 13.20	*t* = −0.77	0.442
Height (cm), Mean ± SD	170.64 ± 7.04	170.23 ± 6.81	171.61 ± 7.66	*t* = −0.69	0.490
Weight (kg), Mean ± SD	75.46 ± 14.47	74.24 ± 12.31	78.36 ± 18.77	*t* = −0.86	0.400
Ratio of deltoid muscle thickness, Mean ± SD, (%)	88.59 ± 15.93	89.93 ± 16.56	85.40 ± 14.26	*t* = 1.01	0.315
Ratio of supraspinatus muscle thickness, Mean ± SD, (%)	88.36 ± 13.29	88.93 ± 13.93	87.01 ± 11.89	*t* = 0.51	0.612
Gender, *n* (%)				χ^2^ = 0.30	0.586
Female	50 (81.97)	34 (79.07)	16 (88.89)		
Male	11 (18.03)	9 (20.93)	2 (11.11)		

*t*, *t*-test, χ^2^, Chi-square test.

SD, Standard deviation.

**Table 4:** Differential analysis of training set.

Variables	Total (*n* = 43)	Group without GHS (*n* = 19)	Group with GHS (*n* = 24)	Statistic	*p*
Age (year), Mean ± SD	55.51 ± 13.24	58.32 ± 14.11	53.29 ± 12.36	*t* = 1.24	0.221
Height (cm), Mean ± SD	170.23 ± 6.81	169.79 ± 4.54	170.58 ± 8.26	*t* = −0.38	0.709
Weight (kg), Mean ± SD	74.24 ± 12.31	75.92 ± 14.21	72.92 ± 10.69	*t* = 0.79	0.433
Ratio of deltoid muscle thickness, Mean ± SD	89.93 ± 16.56	94.61 ± 15.99	86.22 ± 16.37	*t* = 1.69	0.099
Ratio of supraspinatus muscle thickness, Mean ± SD	88.93 ± 13.93	98.54 ± 10.88	81.32 ± 11.17	*t* = −5.08	<0.001
Gender, *n* (%)				χ^2^ = 0.00	1.000
Female	34 (79.07)	15 (78.95)	19 (79.17)		
Male	9 (20.93)	4 (21.05)	5 (20.83)		

*t*, *t*-test; χ^2^, Chi-square test; SD, Standard deviation.

**Figure 2:**
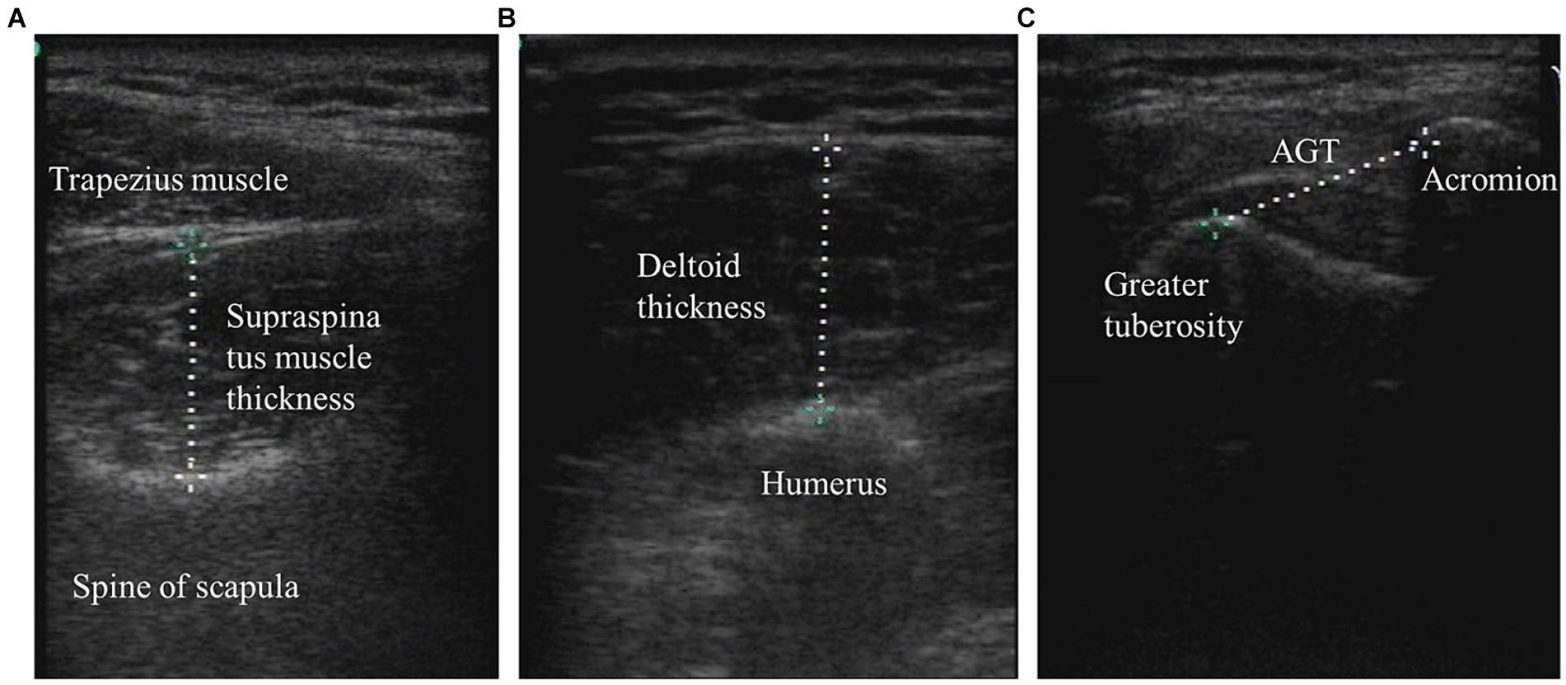
The supraspinatus muscle thickness, deltoid muscle thickness, and AGT were measured by ultrasound imaging. **(A)** Supraspinatus muscle thickness. **(B)** Deltoid thickness; and **(C)** Acromion-greater tuberosity distance.

In the training set, logistic regression analysis was performed on supraspinatus muscle thickness ratio, deltoid muscle thickness ratio, age, height, and weight based on the presence or absence of GHS. According to the obtained odds ratio, the ratio of supraspinatus muscle thickness was statistically significant (OR: 0.80; 95% CI: 0.70 ~ 0.92; *p* < 0.01), and it was an independent factor for evaluating the presence or absence of GHS (Tables 5, 6). Based on the results of the logistic regression analysis, a risk prediction nomogram for shoulder subluxation was constructed (Figure 2).

**Table 5:** Single factor logistic regression results.

Variables	β	S.E	*Z*	*p*	OR (95%CI)
Gender					
Male					1.00 (Reference)
Female	−0.01	0.75	−0.02	0.986	0.99 (0.22 ~ 4.33)
Age	−0.03	0.03	−1.22	0.221	0.97 (0.92 ~ 1.02)
Height	0.02	0.05	0.38	0.701	1.02 (0.93 ~ 1.11)
Weight	−0.02	0.03	−0.80	0.425	0.98 (0.93 ~ 1.03)
Ratio of supraspinatus muscle thickness (%)	−0.22	0.07	−3.25	0.001	0.80 (0.70 ~ 0.92)
Ratio of deltoid muscle thickness (%)	−0.03	0.02	−1.60	0.109	0.97 (0.93 ~ 1.01)

OR, Odds ratio; CI, Confidence interval.

**Table 6:** Multivariate logistic regression results.

Variables	β	S.E	*Z*	*p*	OR (95%CI)
Ratio of supraspinatus muscle thickness	−0.22	0.07	−3.25	0.001	0.80 (0.70 ~ 0.92)

OR, Odds ratio; CI, Confidence interval.

**Figure 3:**
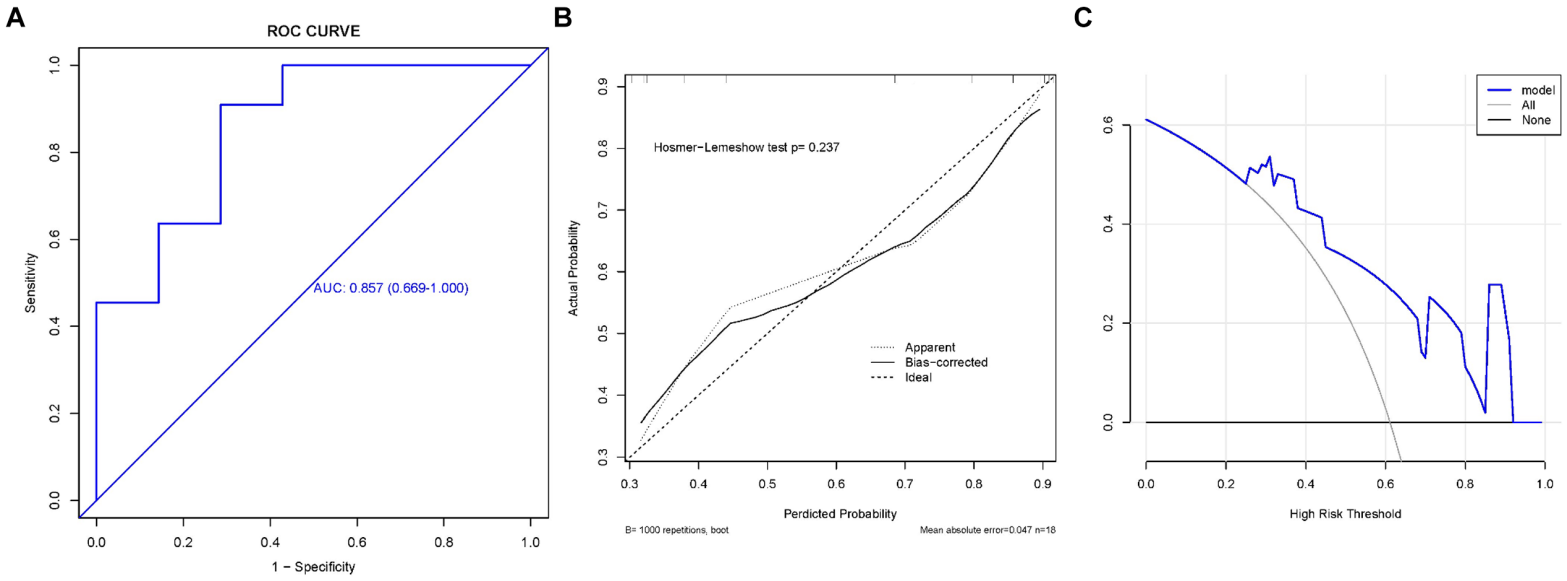
The training set. **(A)** Model ROC curve surface; **(B)** calibration curve of nomogram; and **(C)** decision curve analysis of model. AUC, Area under the curve; GHS, Glenohumeral subluxation; ROC, Receiver operating characteristic.

**Figure 4:**
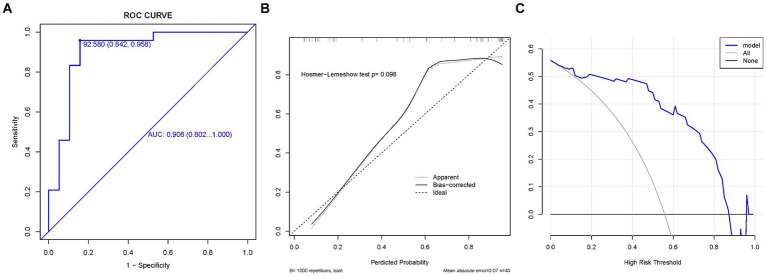
The validation set. **(A)** Model ROC curve surface; **(B)** calibration curve of nomogram; and **(C)** decision curve analysis of m+odel. AUC, Area under the curve; GHS, Glenohumeral subluxation; ROC, Receiver operating characteristic.

We plotted the ROC curves on the training and validation sets, and evaluated their discriminatory power, predictive accuracy, and clinical utility using calibration and decision curves. The ROC curve of the ratio of supraspinatus muscle thickness was calculated using the presence or absence of GHS as the state variable. When evaluating the applicability of the regression model based on the ROC curve, an AUC of 0.906 (95% CI, 0.802–1.000) was found in the training set; meanwhile, the AUC in the validation set was 0.857 (95% CI, 0.669–1.000), indicating good performance (Figures 3, 4). According to the training set ROC curve, the most effective statistical threshold was 93%, with a sensitivity of 84% and a specificity of 96%. The calibration plot showed a close alignment between predicted and observed risks, with a slope close to 1, indicating good consistency between the model predictions and actual results. Finally, the DCA indicated that the threshold probabilities of the training and validation sets of the predictive model were 0–95% and 25–95%, respectively, indicating the strong clinical utility of the line model. In addition, the Hosmer-Lemeshow test showed a high degree of consistency between predicted and actual probabilities, with *p* > 0.05 in both the training and validation sets.

## 4 Discussion and conclusion

This study focused on the relationship between the presence or absence of GHS and the thickness of the supraspinatus muscle. The incidence of subluxation among the study participants was 57%. This was consistent with the incidence of GHS among patients with cerebrovascular disorder hemiplegia, which is 17–81% (7). The results showed that the ratio of supraspinatus muscle thickness in stroke patients was significantly reduced in the group with GHS compared to the group without GHS. The action of the supraspinatus muscle is the abduction of the shoulder joint or humeral head. The contraction of the muscle strengthens the horizontal tension of the shoulder joint capsule. The supraspinatus muscle is in steady contact with the fossa and responds to the load of the joint. Therefore, in patients with cerebrovascular disorder hemiplegia, GHS is common because of the paralysis of the supraspinatus muscle (33). In addition, there was no significant change in deltoid muscle thickness between the group with GHS and the group without GHS. Regardless of whether GHS is present, the thickness of the deltoid muscle on the affected side was lower than that on the unaffected side. Liu et al.’s study showed that the supraspinatus muscle thickness and deltoid muscle thickness on the affected side was significantly smaller than that on the unaffected side in stroke patients with GHS. The results of this study are consistent (20). Although the deltoid and supraspinatus muscles are different in anatomical structure and function, the simultaneous contraction of deltoid and supraspinatus muscles can make the shoulder joint abduct (34, 35). The onset of a stroke causes hemiplegic side muscle strength decreased, muscle tension became imbalanced, and muscle atrophy occurred. These resulted in a simultaneous decrease not only in the function of the deltoid and supraspinatus muscles on the affected side, but also in muscle strength and thickness (20, 31). The supraspinatus muscle is located in the deep layer of the shoulder and is critical for maintaining the stability of the shoulder joint. It ends at the greater tubercle of the humerus and is more sensitive to subluxation (19, 20); therefore, the relationship between supraspinatus muscle and shoulder subluxation was discussed.

In the results of the logistic regression analysis, the only factor selected was the ratio of supraspinatus muscle thickness. Based on the resultant odds ratio, the ratio of supraspinatus muscle thickness was statistically significant. From these results, the ratio of supraspinatus muscle thickness was considered to be useful for GHS evaluation. The AUC of the training set was 0.906, and that of the validation set was 0.857, indicating that the model had good discrimination ability in predicting GHS. When the cut-off value of the supraspinatus muscle thickness difference was 93%, significant results were obtained with a sensitivity of 84% and specificity of 96%. The results suggest that the method employed can be useful in the evaluation of GHS based on relative supraspinatus muscle thickness. If the ratio of the thickness of the supraspinatus muscle between the affected side and the unaffected side is ≤93%, then GHS symptoms may occur, and necessary further diagnosis and rehabilitation measures may be taken. This assessment can help the medical team intervene earlier and improve the treatment effect and the patient’s quality of life. Further, it was discovered that the symptom level was remarkably high. Therefore, the ratio of supraspinatus muscle thickness is a useful criterion for evaluating GHS.

In a post-cerebral vascular injury, the muscles around the shoulder joint initially relax while the alignment of the shoulder blade and humerus changes. At this stage, the dynamic stabilization mechanism does not function correctly; instead, it depends on the static stabilization mechanism. In such cases, the mechanism is pulled excessively by the weight of the upper limbs. Owing to the loss of normal shoulder movement and reduced muscle tension in the upper limbs, the humeral head shifts inferiorly, and the joint capsule is affected by gravity. In these cases, the muscles, tendons, and ligaments are stretched, reducing the ability of the shoulder muscles especially the circumflex tendon plate to hold the humeral head in the joint fossa. These circumstances cause a high risk of subluxation (20). The supraspinatus muscle has a rotational and stabilizing effect. At the beginning of all movements, the tension of the supraspinatus muscle attracts the head of the humerus to the glenoid cavity, maintains its position, and fixes the center of the movement. The supraspinatus muscle is also the suspension muscle when an arm is resting or in a drooping position and when it is holding a bag (36–39). It has been reported that the sub-dislocation of the shoulder joint in patients with cerebrovascular disorder hemiplegia is caused by the weight of the upper limbs, especially the paralysis of the supraspinatus muscle (19, 40–42). Finally, the results of this study showed that, as the ratio of supraspinatus muscle thickness decreases, the risk of GHS increase.

This study had some limitations. First, the sample was taken from a single center and the sample size was small; these features may limit the wide applicability and representativeness of the results. Therefore, multi-center and large sample studies are required to verify the results. Second, this study reviewed patients within 6 months of stroke onset; at this stage, the muscle strength of patients would have changed considerably. Therefore, the cross-sectional design used in this study was not suitable to satisfactorily confirm that this result represents the exact situation of stroke patients. Furthermore, this study did not explore the relationship between shoulder subluxation and other shoulder muscles, or other related variables such as upper limb function and self-care ability; thus, in future, more variables should be included in similar research. Last, although we established the ROC curve model and considered that the ratio of supraspinatus thickness may be an important indicator of shoulder subluxation, this study was a cross-sectional study. Observational studies can rarely yield causal inferences, as models may not be able to exclude all potential confounding factors or reverse causal relationships. Therefore, more longitudinal studies are needed to prove the causal relationship.

This study shows that the ratio of supraspinatus muscle thickness is an important index for evaluating shoulder subluxation in stroke patients. Our model performed well when applied to the training set and independent test set, proving that it has potential for practical clinical applications. It not only provides a new perspective for the mechanism of subluxation, but also important theoretical support and practical guidance for improving rehabilitation strategies for subluxation patients in the future. Moreover, this study underscores that ultrasound imaging remains a useful non-invasive and real-time examination method for the early detection of and interventions for shoulder subluxation. Regular ultrasound examination facilitates the early detection of abnormal shoulder structure or potential signs of subluxation, enabling timely rehabilitative interventions. Such early intervention can not only reduce the risk of shoulder subluxation, but also improve the effect of rehabilitation and, in turn, quality of life. Therefore, the promotion and application of ultrasound imaging technology in the evaluation of shoulder subluxation in patients with hemiplegia has important clinical significance for improving clinical management and the treatment effect.

## Data Availability

The raw data supporting the conclusions of this article will be made available by the authors, without undue reservation.
